# Metagenomics Offers a Big-Picture View of the Diversity and Distribution of Marine Viruses

**DOI:** 10.1371/journal.pbio.0040406

**Published:** 2006-11-07

**Authors:** Mary Hoff

When we think of ocean life, we tend to think of sharks and squid and sea turtles and such. Underpinning these large life forms is a massive but much less conspicuous world of microscopic bacteria and archaea. And existing at an even lower rung, bridging the gap between life and nonlife, are bacteriophage (phage) viruses—minute, self-replicating bundles of biochemicals that alter microorganisms’ genetic material and moderate their communities through predation and parasitism. Although unfathomably tiny, marine bacteriophages are also astoundingly abundant—there are about as many of them in a bucket full of seawater as there are humans on the planet. As a result, they can have a cumulatively huge impact as they individually alter the flow of energy, biomass, and genes through the biosphere.

To get a better picture of the bacteriophage bounty found in marine environments, Florent Angly, Forest Rohwer, and colleagues used metagenomics, an approach that applies genomic techniques to large samples, rather than to individual organisms. By providing a snapshot of the DNA of uncultured viruses in the oceans, metagenomics offers valuable insights into viral diversity, geographical distribution, taxonomic composition, and ecosystem functioning.

The subjects of study were 184 water samples collected from 68 sites over 10 years’ time from four ocean regions: the Sargasso Sea, the Gulf of Mexico, British Columbia coastal waters, and the Arctic Ocean. Each sample was analyzed using a new DNA sequencing technology called pyrosequencing, which makes it possible to obtain a large number of DNA sequences (albeit small ones) at a lower cost than conventional sequencing approaches, to determine the nature of the viral DNA present. The resulting viral metagenomes, or viromes, were compared with a large public database of genomes that have been sequenced, with an “environmental database” consisting of genomes found previously in diverse natural settings, and with an existing database of viral genetic material.

The innovative approach yielded a picture of tremendous diversity in the viral composition of the oceans, with more than 91% of the DNA sequences found differing from the known databases. The genomes found included those of cyanophages, several unusual viruses, and a single-stranded DNA phage—the first of its kind found in abundance in the marine environment—suggesting a unique “marine-ness” in the viral composition of ocean water.

Due to their size and lack of locomotion, viruses are believed to be easily dispersed by marine currents or even sea breezes. The researchers used three statistical approaches to analyze the distribution of marine phages among sampling sites. They found that the distribution among the sample sites was not random and that it tended to differ not only among ocean regions, but also compared with the distribution of land-based viruses. They also showed a correlation between geographic distance and genetic distance between viral species, supporting the contention that the marine virome varies from region to region, even though many species are found at more than one sampling site. Finally, to assess how much the viral makeup of various environments overlaps, the researchers mixed the DNA sequences from the four regions and observed the extent to which fragments with different origins meshed with each other—an indicator of the similarity of the viromes. A simulation of this data suggested that the differences among the regions was mostly explained by variations in relative abundance of the predominant viral species, rather than by the range of viruses present at each site. This supports the saying that “Everything is everywhere, but, the environment selects.”

**Figure:**
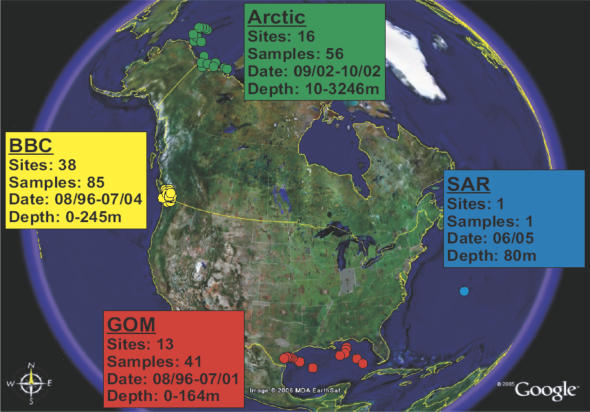
The authors used metagenomics to analyze the “viromes” of oceanic viruses and shed light on their diversity, distribution, and ecosystem impact in four ocean regions around the world.

So, how diverse is the viral makeup of the marine environment? Samples taken off the British Columbia coast were the most genetically diverse—not surprising, since an upwelling in the area offers a nutrient-rich environment for supporting a wide range of life forms upon which viruses depend. The other three samples showed increasing diversity with decreasing latitude, a trend that parallels previous findings from terrestrial ecosystems. Extrapolating from their observations, the researchers predicted that the world’s oceans hold a few hundred thousand broadly distributed viral species, with some species-rich regions likely harboring the majority of these species.

In addition to analyzing their results, the researchers commented that they obtained and combined multiple samples in space and time from all but the Sargasso Sea site, because they thought this would provide the best approximation of the actual meta-viral profiles. The data analysis of the single Sargasso Sea sample, however, led them to conclude that individual samples at the other sites might have led to equally representative results. Such a sampling approach, they noted, would yield additional benefits in the form of opportunities to explore spatio-temporal gradations not discernable using the integrative sampling approach. Other changes they proposed to further expand the usefulness of viral metagenomic analysis include expanding sampling capability to include large DNA viruses and finding a way to include RNA viruses. The researchers are looking forward to future studies that will further characterize the viral makeup of the oceans and other unsequenced environments, including ones that explore the nature and the implications for ecosystems of marine viruses’ relationship with their microbial hosts.

